# Overall and disease‐specific survival of Hodgkin lymphoma survivors who subsequently developed gastrointestinal cancer

**DOI:** 10.1002/cam4.1922

**Published:** 2018-12-27

**Authors:** Lisanne S. Rigter, Michael Schaapveld, Cecile P. M. Janus, Augustinus D. G. Krol, Richard W. M. van der Maazen, Judith Roesink, Josee M. Zijlstra, Gustaaf W. van Imhoff, Philip M. P. Poortmans, Max Beijert, Pieternella J. Lugtenburg, Otto Visser, Petur Snaebjornsson, Anna M. van Eggermond, Berthe M. P. Aleman, Flora E. van Leeuwen, Monique E. van Leerdam

**Affiliations:** ^1^ Department of Gastroenterology Netherlands Cancer Institute Amsterdam The Netherlands; ^2^ Division of Epidemiology Netherlands Cancer Institute Amsterdam The Netherlands; ^3^ Department of Radiation Oncology, Erasmus MC Cancer Institute University Medical Center Rotterdam The Netherlands; ^4^ Department of Clinical Oncology Leiden University Medical Centre Leiden The Netherlands; ^5^ Department of Radiation Oncology Radboud University Medical Center Nijmegen The Netherlands; ^6^ Department of Radiation Oncology University Medical Center Utrecht Utrecht The Netherlands; ^7^ Department of Hematology VU University Medical Center Amsterdam The Netherlands; ^8^ Department of Hematology University of Groningen, University Medical Center Groningen The Netherlands; ^9^ Department of Radiation Oncology University Medical Center Groningen Groningen The Netherlands; ^10^ Department of Hematology Erasmus MC Cancer Institute, University Medical Center Rotterdam The Netherlands; ^11^ Registration and Research, Comprehensive Cancer Center The Netherlands Utrecht The Netherlands; ^12^ Department of Pathology Netherlands Cancer Institute Amsterdam The Netherlands; ^13^ Department of Radiation Oncology Netherlands Cancer Institute Amsterdam The Netherlands

**Keywords:** gastrointestinal cancer, Hodgkin lymphoma, second malignancy, survival

## Abstract

**Background:**

Hodgkin lymphoma (HL) survivors have an increased risk of gastrointestinal (GI) cancer. This study aims to evaluate whether survival of patients who survived HL and developed GI cancer differs from survival of first primary GI cancer patients.

**Methods:**

Overall and cause‐specific survival of GI cancer patients in a HL survivor cohort (GI‐HL, N = 104, including esophageal, gastric, small intestinal, and colorectal cancer) was compared with survival of a first primary GI cancer patient cohort (GI‐1, N = 1025, generated by case matching based on tumor site, gender, age, and year of diagnosis). Cox proportional hazards regression was used for survival analyses. Multivariable analyses were adjusted for GI cancer stage, grade of differentiation, surgery, radiotherapy, and chemotherapy.

**Results:**

GI‐HL cancers were diagnosed at a median age of 54 years (interquartile range 45‐60). No differences in tumor stage or frequency of surgery were found. GI‐HL patients less often received radiotherapy (8% vs 23% in GI‐1 patients, *P < *0.001) and chemotherapy (28% vs 41%, *P = *0.01) for their GI tumor. Compared with GI‐1 patients, overall and disease‐specific survival of GI‐HL patients was worse (univariable hazard ratio (HR) 1.30, 95% confidence interval (CI) 1.03‐1.65, *P = *0.03; and HR 1.29, 95% CI 1.00‐1.67, *P = *0.049, respectively; multivariable HR 1.33, 95% CI 1.05‐1.68, *P = *0.02; and HR 1.33, 95% CI 1.03‐1.72, *P = *0.03, respectively).

**Conclusions:**

Long‐term overall and disease‐specific survival of GI cancer in HL survivors is worse compared with first primary GI cancer patients. Differences in tumor stage, grade of differentiation, or treatment could not explain this worse survival.

## 1 INTRODUCTION

Hodgkin lymphoma (HL) survivors are at increased risk of developing second malignancies, which are a major cause for morbidity and mortality.^1^, ^2^, ^3^, ^4^ Compared with the general population, the risk of developing gastrointestinal (GI) cancer is approximately 5‐fold higher in HL survivors.^3^, ^4^, ^5^, ^6^, ^7^, ^8^, ^9^ This risk remains elevated up to 40 years after HL and is strongly related to HL treatment.^4^ Both exposure to radiotherapy and alkylating agents, such as procarbazine or dacarbazine, have been associated with the development of GI cancers.^3^, ^4^, ^5^, ^6^, ^7^, ^9^, ^10^, ^11^

A few studies suggest a difference in clinical and histopathological characteristics of GI cancer in HL survivors compared with first primary GI cancer.^12^, ^13^, ^14^ To our knowledge, only one previous study examined survival of GI cancer in HL survivors and reported a worse overall survival in subgroups of HL survivors compared with first primary GI cancer patients, that is, those diagnosed with TNM stage IIB‐IV colon cancer and a small group (N = 8) of TNM stage I gastric cancer.^13^ No differences in disease‐specific survival were found.

The cause of the reported reduced overall survival of GI cancers in HL survivors remained unknown. Less favorable survival might be due to differences in (HL treatment‐induced) carcinogenesis leading to differences in GI tumor characteristics, or to adaptation of GI cancer treatment due to the previous treatment for HL. Furthermore, increased risks of competing causes of death, such as other malignancies or cardiovascular disease, might play a role.^15^, ^16^, ^17^

In view of the reported worse overall survival of GI cancer in HL survivors and its unknown etiology, we designed this study to evaluate overall and cause‐specific survival of GI cancer in HL survivors.

## 2 PATIENTS AND METHODS

### 2.1 Study design

This study compared overall and cause‐specific survival of esophageal, gastric, small intestinal, and colorectal cancer in a HL survivor cohort (GI‐HL) with survival of a population‐based cohort of first primary GI cancer patients (GI‐1).

In a Dutch multicenter cohort of HL patients who survived at least 5 years after primary treatment (N = 2996), 121 GI‐HL patients with carcinomas of the esophagus, stomach, small intestine, or colorectum were identified. Data on HL patients, diagnosed in the period 1965‐2000 and between 15 and 50 years of age at HL diagnosis, were collected as previously described.^4^, ^15^, ^17^ In short, data collection comprised detailed HL treatment data and information on second cancers, using medical records, by responses to questionnaires sent to general practitioners and linkage with the Netherlands Cancer Registry (NCR, from 1989 onwards).^4^ Finally, a total of 104 GI‐HL patients were used for analyses (Figure S1).

For each GI‐HL cancer, 10 matched controls of the Dutch general population with a GI‐1 cancer were identified, based on the following criteria: gender, no prior diagnosis of invasive tumors, tumor location (esophagus, stomach, small intestine, or colorectum), year of diagnosis (closest proximity, maximum of 5 years difference), and age at diagnosis (closest proximity, maximum of 3 years difference). For three GI‐HL patients, it was not possible to obtain 10 matched GI‐1 patients because of the young age at diagnosis. Subsequently, data on GI cancer characteristics, treatment, and follow‐up were collected for both GI‐HL and GI‐1 patients.

From Statistics Netherlands (CBS), we obtained information on the cause of death, which was categorized into GI cancer of interest or other causes, including unknown causes. As all data were processed and analyzed anonymously, this study was exempt from review by the Institutional Review Board.

### 2.2 Statistical analyses

Patient and tumor characteristics of GI‐HL and GI‐1 patients were compared using chi‐square, Fishers’ exact, or Mann‐Whitney *U* tests. Overall survival and cause‐specific survival were presented using the Kaplan‐Meier method. Cause‐specific survival was divided into disease‐specific survival, related to the GI cancer of interest, and survival related to other causes of death (using GI cancer‐related death as a censoring event).

In 12 out of 104 GI‐HL patients, the HL‐GI tumor was not the first diagnosis of a malignancy after HL. Since these other primary tumors or their treatment might affect survival, these 12 patients and their matched controls were excluded from further survival analyses (Table S1). Thus, 92/104 GI‐HL tumors and their 911 matched controls were included in Cox proportional hazards regression models. We evaluated the effect of patient‐related and tumor‐related characteristics on the survival difference between GI‐HL and GI‐1 patients, that is on the HR associated with GI cancer in HL survivors (GI‐1 patients are included in the model as the reference population). We added each characteristic to the regression model and evaluated the influence of this characteristic on the survival difference between GI‐HL and GI‐1 patients, for example the hazard ratio. In case of a >10% change in the hazard ratio for death associated with the grouping variable (eg, GI‐HL vs GI‐1), this characteristic was considered to have a substantial influence on the survival difference between the groups. We also assessed disease‐specific mortality while treating other causes of death as a competing risk.

Analyses were performed using IBM SPSS Statistics 22 and STATA version 14 (Armonk, New York).

## 3 RESULTS

### 3.1 General description and comparison of GI‐HL and GI‐1 patients

GI‐HL cancers were diagnosed at a median age of 54 years (interquartile range (IQR) 45‐60). The majority occurred in males (67%). Patients were diagnosed with HL at a median age of 30 years (interquartile range (IQR) 22‐41, Table S1). Median year of HL diagnosis was 1981 (range 1966‐2000). In 53/104 (51%) patients, HL had been treated with both radiotherapy and procarbazine‐containing chemotherapy and 43/104 (41%) patients had been treated for a HL recurrence.

Due to the matching procedure, GI‐HL cancers were not different from GI‐1 cancers with respect to gender, age at diagnosis, and TNM stage (Table 1). GI‐HL patients were less frequently treated for their GI tumor with radiotherapy (8% vs 23% in GI‐1 patients, *P < *0.001) or chemotherapy (29% vs 41%, *P = *0.01). Compared with GI‐1 tumors, GI‐HL tumors were treated more frequently with surgery alone and less frequently with combined modality treatment that included radiotherapy or chemotherapy (*P = *0.005, Table 1).

**Table 1:** Characteristics of gastrointestinal cancer in Hodgkin lymphoma survivors and first primary gastrointestinal cancer patients

GI cancer characteristic	Gastrointestinal cancer			Esophageal cancer			Gastric cancer			Colorectal cancer		
	GI‐HL (N = 104)	GI‐1 (N = 1025)	*P* value	GI‐HL (N = 30)	GI‐1 (N = 287)	*P* value	GI‐HL (N = 34)	GI‐1 (N = 338)	*P* value	GI‐HL (N = 38)	GI‐1 (N = 380)	*P* value
	n (%)	n (%)		n (%)	n (%)		n (%)	n (%)		n (%)	n (%)	
Age Median (IQR)	54 (45‐60)	54 (45‐60)	0.82	54 (45‐59)	54 (46‐59)	0.67	46 (36‐55)	47 (36‐54)	0.98	56 (49‐61)	56 (49‐61)	1.00
Gender												
Male	70 (67)	698 (68)	0.87	18 (60)	180 (63)	0.77	22 (65)	218 (64)	1.00	29 (76)	290 (76)	1.00
Female	34 (33)	327 (32)		12 (40)	107 (37)		12 (35)	120 (36)		9 (24)	90 (24)	
Morphology category												
Adenocarcinoma	81 (78)	900 (88)	<0.001	10 (33)	167 (58)	0.005	32 (94)	335 (99)	0.07	38 (100)	378 (100)	0.65
Squamous cell carcinoma	18 (17)	117 (11)		17 (57)	115 (40)		1 (3)	2 (1)		‐	‐	
Other carcinoma^a^	5 (5)	8 (1)		3 (10)	5 (2)		1 (3)	1 (<1)		0 (0)	2 (<1)	
TNM stage												
I	12 (12)	124 (12)	0.79	2 (7)	21 (7)	0.23	8 (24)	49 (14)	0.52	2 (5)	54 (14)	0.11
II	24 (23)	202 (20)		4 (13)	40 (14)		5 (15)	50 (15)		14 (37)	109 (29)	
III	26 (25)	253 (25)		14 (47)	85 (30)		6 (18)	56 (17)		6 (16)	103 (27)	
IV	35 (34)	387 (38)		6 (20)	110 (38)		13 (38)	162 (48)		15 (39)	108 (28)	
Unknown	7 (7)	59 (6)		4 (13)	31 (11)		2 (6)	21 (6)		1 (3)	6 (2)	
Grade of differentiation												
Well/low grade	8 (8)	36 (4)	0.005	0 (0)	10 (4)	0.21	1 (3)	4 (1)	0.02	7 (18)	21 (6)	0.04
Moderate/intermediate	34 (33)	340 (33)		10 (33)	78 (27)		6 (18)	53 (16)		18 (47)	204 (54)	
Poor/high	22 (21)	338 (33)		6 (20)	104 (36)		11 (32)	171 (51)		5 (13)	55 (14)	
Undifferentiated/anaplastic	4 (4)	10 (1)		1 (3)	4 (1)		3 (9)	5 (1)		0 (0)	1 (<1)	
Unknown	36 (35)	301 (29)		13 (43)	91 (32)		13 (38)	105 (31)		8 (21)	99 (26)	
Surgery												
No	37 (36)	396 (39)	0.54	16 (53)	179 (62)	0.33	13 (38)	162 (48)	0.28	7 (18)	49 (13)	0.34
Yes	67 (64)	629 (61)		14 (47)	108 (38)		21 (62)	176 (52)		31 (82)	331 (87)	
Radiotherapy												
No	96 (92)	785 (77)	<0.001	24 (80)	173 (60)	0.03	34 (100)	313 (93)	0.15	36 (95)	280 (74)	0.004
Yes	8 (8)	240 (23)		6 (20)	114 (40)		0 (0)	25 (7)		2 (5)	100 (26)	
Chemotherapy												
No	75 (72)	608 (59)	0.01	26 (87)	165 (58)	0.001	26 (77)	204 (60)	0.07	22 (58)	226 (60)	0.85
Yes	29 (28)	417 (41)		4 (13)	122 (43)		8 (24)	134 (40)		16 (42)	154 (41)	
Treatment category												
No treatment	18 (17)	153 (15)	0.005	10 (33)	65 (23)	0.015	6 (18)	69 (20)	0.054	2 (5)	16 (4)	0.39
Surgery only	51 (49)	348 (34)		12 (40)	59 (21)		20 (59)	125 (37)		18 (47)	154 (41)	
Surgery & RT and/or CT	16 (15)	281 (27)		2 (7)	49 (17)		1 (3)	51 (15)		13 (34)	177 (47)	
RT and/or CT only	19 (18)	243 (24)		6 (20)	114 (40)		7 (21)	93 (28)		5 (13)	33 (9)	

Gastrointestinal cancers include two GI‐HL small intestinal cancers and their matched GI‐1 controls.

GI‐HL, gastrointestinal cancer in Hodgkin lymphoma survivors; GI‐1, first primary gastrointestinal cancer patients; RT, radiotherapy; CT, chemotherapy.

a Defined as neuroendocrine carcinomas, large cell carcinomas, undifferentiated carcinomas, anaplastic carcinomas, or unspecified carcinomas.

### 3.2 Gastrointestinal cancer: overall survival

**Figure 1:**
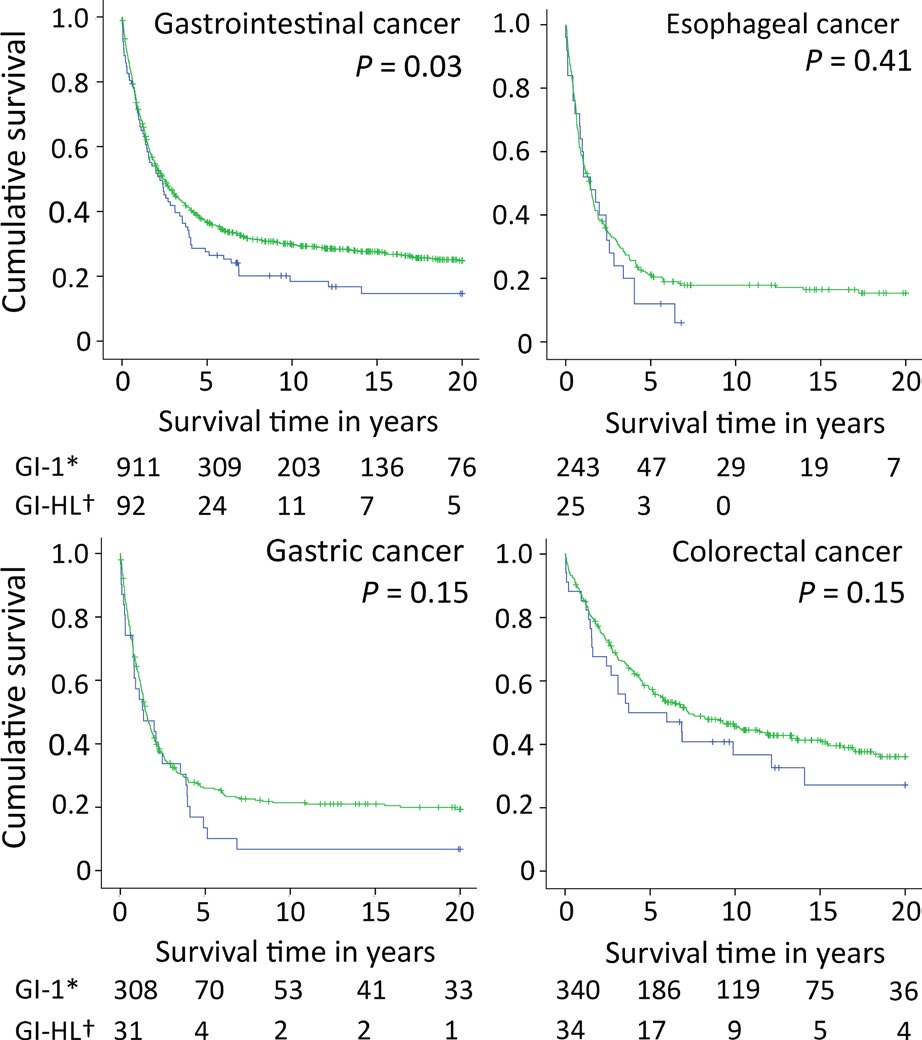
Overall survival of gastrointestinal cancer in Hodgkin lymphoma survivors (GI‐HL, blue) compared with first primary gastrointestinal cancer patients (GI‐1, green). *First primary gastrointestinal cancer patients; green line, number of cases at risk. ^†^Gastrointestinal cancer in Hodgkin lymphoma survivors; blue line, number of cases at risk

Overall survival of 104 GI‐HL patients was worse than that of 1025 GI‐1 patients (hazard ratio (HR) 1.27, 95% confidence interval (CI) 1.01‐1.58, *P = *0.037). After exclusion of 12 GI‐HL patients with a third primary gastrointestinal tumor, overall survival in the remaining 92 GI‐HL patients was worse compared with 911 GI‐1 patients (HR 1.30, 95% CI 1.03‐1.65, *P = *0.028, Table S1, Figure 1, Table 2).

**Table 2:** Overall survival of gastrointestinal cancer in Hodgkin lymphoma survivors compared with first primary gastrointestinal cancer patients

Characteristic	Gastrointestinal cancer		Esophageal cancer		Gastric cancer		Colorectal cancer	
	GI‐HL (N = 92) % (95% CI)	GI‐1 (N = 911 % (95% CI)	GI‐HL (N = 25) % (95% CI)	GI‐1 (N = 243) % (95% CI)	GI‐HL (N = 31) % (95% CI)	GI‐1 (N = 308) % (95% CI)	GI‐HL (N = 34) % (95% CI)	GI‐1 (N = 340) % (95% CI)
5‐y survival	28 (18‐37)	37 (34‐40)	12 (0‐25)	21 (16‐27)	13 (1‐26)	26 (21‐31)	50 (33‐67)	57 (52‐63)
10‐y survival	18 (10‐27)	30 (27‐33)	6 (0‐16)	18 (13‐23)	7 (0‐16)	21 (17‐26)	37 (20‐53)	46 (40‐51)
15‐y survival	15 (7‐23)	28 (25‐31)	6 (0‐16)	16 (12‐21)	7 (0‐16)	21 (16‐26)	27 (10‐44)	41 (36‐47)

GI‐HL vs GI‐1 (ref)	HR (95% CI)	*P* value	HR (95% CI)	*P* value	HR (95% CI)	*P* value	HR (95% CI)	*P* value
Univariable	1.30 (1.03‐1.65)	0.03	1.20 (0.79‐1.85)	0.41	1.33 (0.91‐1.96)	0.15	1.36 (0.90‐2.06)	0.15
Multivariable, including								
Tumor characteristics^a^	1.39 (1.10‐1.76)	0.006						
Treatment characteristics^b^	1.32 (1.04‐1.68)	0.02						
Tumor + treatment^c^	1.33 (1.05‐1.68)	0.02						
Tumor subsite^d^			1.15 (0.74‐1.79)	0.54	1.71 (1.14‐2.55)	0.009	1.29 (0.85‐1.96)	0.24

GI‐HL, gastrointestinal cancer in Hodgkin lymphoma survivors; GI‐1, first primary gastrointestinal cancer patients; HR, hazard ratio; 95% CI, 95% confidence interval.

a Cox proportional hazards regression model adjusted for dichotomized variables TNM stage (I/II vs III/IV), grade of differentiation (well/moderate vs poor/undifferentiated), and tumor location (esophagus/stomach vs small intestine/colorectum).

b Cox proportional hazards regression model adjusted for surgery, radiotherapy, chemotherapy.

c Cox proportional hazards regression model adjusted for dichotomized variables TNM stage, grade of differentiation surgery, radiotherapy, chemotherapy.

d Cox proportional hazards regression model adjusted for tumor subsite: esophageal cancer: upper vs other, gastric: antrum/pylorus vs other, colorectal cancer: colon vs rectum.

In a multivariable model, adjusted for tumor characteristics (TNM stage, grade of differentiation, tumor location), the difference between GI‐HL and GI‐1 patients remained present (HR 1.33, 95% CI 1.05‐1.68, *P = *0.020). This difference also remained present after adjustment for treatment characteristics (surgery, radiotherapy, chemotherapy) and after adjustment for both tumor and treatment characteristics (HR 1.32, 95% CI 1.04‐1.68, *P = *0.02; and HR 1.33, 95% CI 1.05‐1.68, *P = *0.02, respectively).

### 3.3 Gastrointestinal cancer: cause‐specific survival

Disease‐specific survival was worse in GI‐HL patients than in GI‐1 patients (HR 1.29, 95% CI 1.00‐1.67, *P = *0.049, Table 3). Mortality from other causes appeared to be nonsignificantly higher in GI‐HL patients compared with GI‐1 patients (HR 1.44, 95% CI 0.81‐2.56, *P = *0.22).

**Table 3:** Cause‐specific cumulative mortality from gastrointestinal cancer in Hodgkin lymphoma survivors and first primary gastrointestinal cancer patients

Cumulative mortality	Gastrointestinal cancer		Esophageal cancer		Gastric cancer		Colorectal cancer	
	GI‐HL (N = 92) % (95% CI)	GI‐1 (N = 911) % (95% CI)	GI‐HL (N = 25) % (95% CI)	GI‐1 (N = 243) % (95% CI)	GI‐HL (N = 31) % (95% CI)	GI‐1 (N = 308) % (95% CI)	GI‐HL (N = 34) % (95% CI)	GI‐1 (N = 340) % (95% CI)
5‐y mortality								
GI cancer	66 (55‐75)	56 (53‐59)	79 (57‐91)	74 (68‐79)	81 (62‐91)	65 (59‐70)	44 (27‐60)	35 (30‐41)
Other causes of death	7 (3‐13)	7 (6‐9)	8 (1‐23)	5 (3‐8)	6 (1‐19)	9 (6‐13)	6 (1‐17)	7 (5‐10)
10‐y mortality								
GI cancer	72 (62‐80)	61 (57‐64)	85 (62‐95)	76 (70‐81)	87 (69‐95)	69 (63‐74)	50 (33‐66)	42 (37‐48)
Other causes of death	9 (4‐17)	10 (8‐12)	8 (1‐23)	7 (4‐10)	6 (1‐19)	10 (9‐14)	13 (4‐27)	12 (9‐16)
15‐y mortality								
GI cancer	72 (62‐80)	61 (58‐65)	85 (62‐95)	76 (70‐81)	87 (69‐95)	69 (63‐74)	50 (33‐66)	45 (39‐50)
Other causes of death	13 (7‐22)	11 (9‐13)	8 (1‐23)	8 (5‐12)	6 (1‐19)	10 (9‐14)	22 (9‐40)	14 (10‐18)

Disease‐specific GI‐HL vs GI‐1 (ref)	HR^a^ (95% CI)	*P* value	HR^a^ (95% CI)	*P* value	HR^a^ (95% CI)	*P* value	HR^b^ (95% CI)	*P* value
Univariable	1.29 (1.00‐1.67)	0.049	1.17 (0.75‐1.84)	0.49	1.43 (0.95‐2.13)	0.08	1.27 (0.77‐2.10)	0.35
Multivariable, including								
Treatment characteristics^a^	1.33 (1.03‐1.72)	0.03						
Tumor + treatment^b^	1.33 (1.03‐1.72)	0.03						
Tumor subsite^c^			1.11 (0.70‐1.76)	0.67	1.80 (1.19‐2.74)	0.006	1.11 (0.66‐1.86)	0.70
Other causes of death								
Univariable	1.44 (0.81‐2.56)	0.22	1.60 (0.36‐7.06)	0.53	1.02 (0.31‐3.34)	0.97	1.61 (0.76‐3.38)	0.21

GI‐HL, gastrointestinal cancer in Hodgkin lymphoma survivors; GI‐1, first primary gastrointestinal cancer patients; HR, hazard ratio; 95% CI, 95% confidence interval.

Cumulative mortality was calculated using competing risk analyses.

a Cox proportional hazards regression model adjusted for surgery, radiotherapy, chemotherapy.

b Cox proportional hazards regression model adjusted for dichotomized variables TNM stage, grade of differentiation, surgery, radiotherapy, chemotherapy.

c Cox proportional hazards regression model adjusted for tumor subsite: esophageal cancer: upper vs other, gastric: antrum/pylorus vs other, colorectal cancer: colon vs rectum.

In a multivariable model adjusted for treatment characteristics, disease‐specific survival remained worse in GI‐HL patients than in GI‐1 patients (HR 1.33, 95% CI 1.03‐1.72, *P = *0.03). After adjustment for both tumor characteristics and treatment characteristics, this survival difference also remained present (HR 1.33, 95% CI 1.03‐1.72, *P = *0.03).

### 3.4 Gastrointestinal cancer subsites

**Figure 2:**
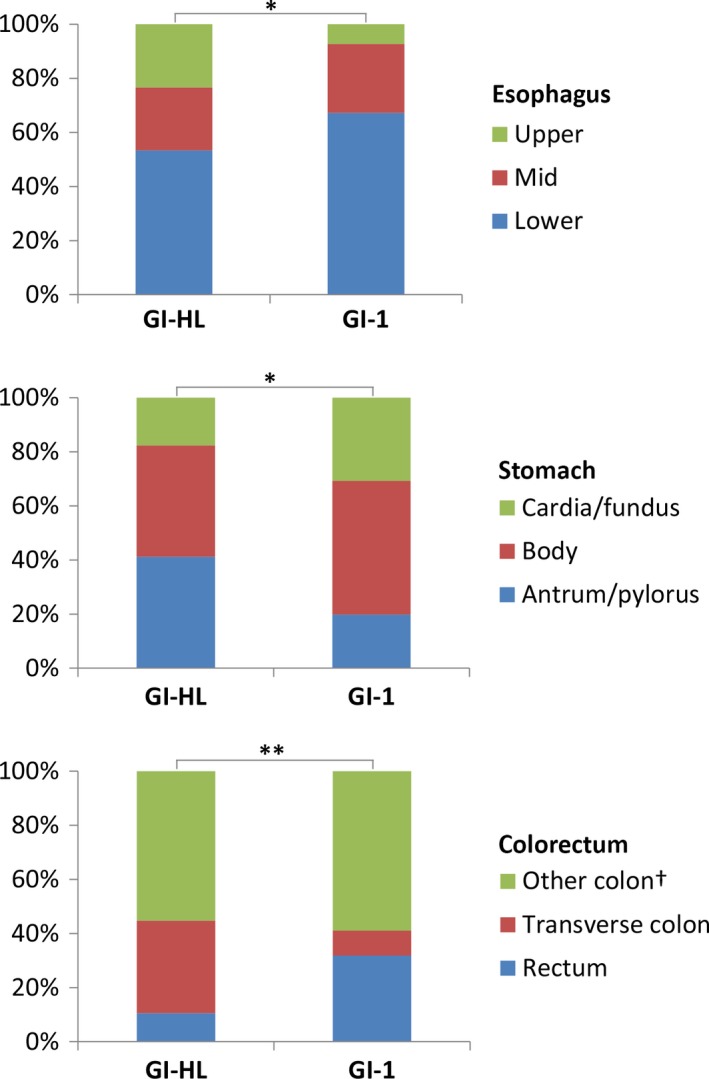
Subsite of gastrointestinal cancer in Hodgkin lymphoma survivors and first primary gastrointestinal cancer patients. Both midesophagus and stomach body contain overlapping or unspecified locations. GI‐HL, gastrointestinal cancer in Hodgkin lymphoma survivors; GI‐1, first primary gastrointestinal cancer patients. *P = 0.01; **P < 0.001; ^†^including cecum, ascending, descending, sigmoid, overlapping, colon not otherwise specified (transverse colon includes the hepatic and splenic flexure)

Within GI cancer subsites, locations of GI‐HL cancers differed significantly from locations of GI‐1 cancers (Figure 2). Overall survival and disease‐specific survival were not significantly different in GI‐HL esophageal cancer patients compared with GI‐1 patients (HR 1.20, 95% CI 0.79‐1.85, *P = *0.41; and HR 1.17, 95% CI 0.75‐1.84, *P = *0.49, respectively, Figure 1, Table 2).

In GI‐HL gastric cancer patients, there was a trend toward worse overall and disease‐specific survival compared with GI‐1 patients (HR 1.33, 95% CI 0.91‐1.96, *P = *0.15; and HR 1.43, 95% CI 0.95‐2.13, *P = *0.08, respectively, Figure 1, Tables 2 and 3). In several multivariable models, the overall and disease‐specific survival difference between GI‐HL gastric cancer patients and GI‐1 patients substantially increased (>10% change in HR of the grouping variable GI‐HL vs GI‐1 patients; disease‐specific survival adjusted for subsite (antrum/pylorus vs other), HR 1.80, 95% CI 1.19‐2.74, *P = *0.006; adjusted for stage, HR 1.66, 95% CI 1.11‐2.49, *P = *0.01; adjusted for surgery, HR 2.00, 95% CI 1.33‐3.01, *P = *0.001, Table S2). None of the evaluated characteristics decreased the survival difference, so none of these characteristics could explain the observed difference in survival.

When comparing GI‐HL colorectal cancer patients with GI‐1 patients, overall survival and disease‐specific survival were not significantly different (HR 1.36, 95% CI 0.90‐2.06 *P = *0.15; and HR 1.27, 95% CI 0.77‐2.10, *P = *0.35, respectively). After adjustment for location either in colon or rectum, disease‐specific survival differences between GI‐HL patients and GI‐1 patients became substantially smaller (HR 1.11, 95% CI 0.66‐1.86, *P = *0.70, Table S2).

## 4 DISCUSSION

Our study is the first to demonstrate both a worse overall survival and disease‐specific survival of GI‐HL patients compared with survival of GI‐1 patients. Although some differences in GI tumor characteristics and treatment were present between GI‐HL patients and GI‐1 patients, none of these characteristics offered sufficient explanation for the survival differences. Mortality from other causes was not significantly higher in GI‐HL patients, but this could be due to a lack of statistical power. However, a higher rate of morbidity may have influenced the efficacy of GI‐HL treatment. In addition, a different pathogenesis of therapy‐related GI cancer may affect the efficacy of GI‐HL treatment, resulting in worse survival.

A difference in carcinogenesis has been suggested only for therapy‐related colorectal cancer diagnosed in HL survivors, as these tumors are more frequently microsatellite instable due to somatic mutations in mismatch repair genes.^14^ In therapy‐related esophageal cancer compared with sporadic cancer, no difference in frequency of microsatellite instability or loss of heterozygosity was found.^12^ To our knowledge, no data are available for therapy‐related gastric cancer and therapy‐related small bowel cancer.

A second important finding of our study is that GI‐HL patients were treated differently compared with GI‐1 patients. GI‐HL patients were more frequently treated with surgery alone, and combined modality treatments were less frequently given. Probably due to prior HL treatment, radiotherapy and chemotherapy are given less frequently for GI‐HL patients, either as a result of dosage limitations or comorbidity.^15^, ^16^ Additionally, the differences in treatment may partially result from the distribution of GI cancer subsites in GI‐HL patients, as these were for example less frequently located in the rectum. Previous studies also reported that therapy‐related GI cancers are more frequently located within irradiation fields.^10^, ^13^, ^18^ Surprisingly, the observed treatment differences did not explain the worse survival. Unfortunately, we did not have detailed data on GI cancer treatment regimens (eg, sequences of treatment, type of chemotherapy).

The only previous, comparable study performed used a similar study design but had somewhat different results.^13^ They found a worse overall survival for HL survivors with TNM stage I gastric cancer (N = 8) and with TNM stage IIB‐IV colorectal cancer (N = 70) compared with a significantly older population cohort with primary GI cancers. This study did not show a difference in overall survival for other stage subgroups or in disease‐specific survival. In addition, our methods of patient selection differed from Youn et al As GI‐HL cancer is diagnosed at a relatively young age, we deliberately generated our population‐based comparison cohort with primary GI cancers by matching on age at diagnosis, and additionally on year of diagnosis and gender. We excluded GI‐HL patients with a second malignancy between HL and GI‐HL from survival analyses to increase comparability with the GI‐1 population. This selection method may have caused a decrease in mortality from other causes in the GI‐HL population, resulting in a more comparable mortality from non‐GI cancer‐related causes for GI‐HL patients and GI‐1 patients.

The selection procedure of the population‐based controls is one of the strengths of this study. Also, this is the first study with sufficient and long‐term follow‐up data to demonstrate a worse overall and disease‐specific survival in patients who survived HL and developed GI cancer and to provide data that excluded several possible etiologic factors.

The survival differences were, however, not large and the power was insufficient to confirm differences in survival between GI‐HL and GI‐1 patients for GI cancer subsites, or for specific HL treatment exposure subgroups (as the majority received combination treatments for HL, which limits statistical power). An additional limitation was the absence of information on other factors associated with GI cancer risk, such as family history and smoking status.

As HL survivors have an increased incidence of GI malignancies, and a slightly worse survival, treating physicians should focus on GI cancer awareness and prevention. Personalized surveillance programs should be developed for this purpose. Our research group is currently performing a multicenter cohort study on a first surveillance colonoscopy in HL survivors.^19^ (Dutch Trial Registry NTR4961) Additionally, further research is necessary to evaluate therapy‐related GI carcinogenesis, as differences compared with sporadic carcinogenesis may have consequences for the clinical approach, such as surveillance technique and interval.

In current clinical practice, decision‐making about curative HL treatment involves the balance of disease control and the risk of long‐term side effects. Due to the increased GI cancer risk associated with radiotherapy and procarbazine, and the associated increased mortality from GI cancer, the indication for the BEACOPP (including procarbazine) regimen should involve careful consideration and radiation fields should be limited.^20^

In conclusion, overall and disease‐specific survival of GI cancer patients is slightly worse in HL survivors compared with first primary GI cancer patients. Differences in tumor stage, grade of differentiation, treatment, or mortality from other causes could not explain the worse survival of GI cancer in HL survivors. As such, this may be explained by a worse treatment response due to HL‐related comorbidities or due to a different pathogenesis of therapy‐related GI cancer.

## CONFLICT OF INTEREST

None declared.
