# Supplementary material for: Overall and disease‐specific survival of Hodgkin lymphoma survivors who subsequently developed gastrointestinal cancer
Source: Cancer Med. 2018 Dec 27;8(1):190–9. doi: 10.1002/cam4.1922 (PMC6346242; doi:10.1002/cam4.1922)
Supplement: Supplementary file 1 [file CAM4-8-190-s001.pdf]

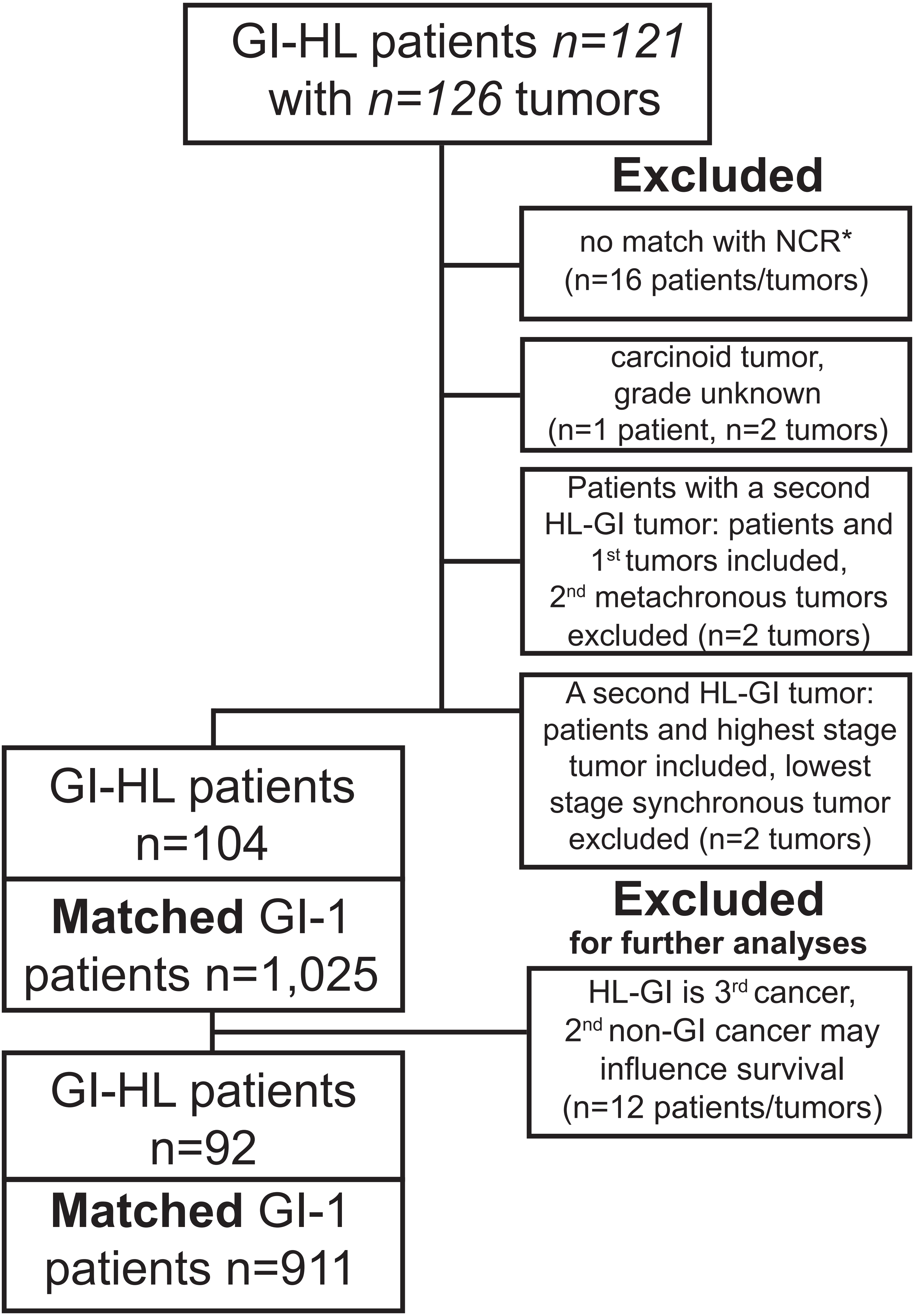
GI-HL patients *n=121*
with *n=126* tumors
Excluded
no match with NCR*
(n=16 patients/tumors)
carcinoid tumor,
grade unknown
(n=1 patient, n=2 tumors)
Patients with a second HL-GI tumor: patients and 1st tumors included, 2nd metachronous tumors excluded (n=2 tumors)
A second HL-GI tumor: patients and highest stage tumor included, lowest stage synchronous tumor excluded (n=2 tumors)
GI-HL patients
n=104
Matched GI-1
patients n=1,025
Excluded
for further analyses
HL-GI is 3rd cancer, 2nd non-GI cancer may influence survival
(n=12 patients/tumors)
GI-HL patients
n=92
Matched GI-1
patients n=911
