# Supplementary material for: Overall and disease‐specific survival of Hodgkin lymphoma survivors who subsequently developed gastrointestinal cancer
Source: Cancer Med. 2018 Dec 27;8(1):190–9. doi: 10.1002/cam4.1922 (PMC6346242; doi:10.1002/cam4.1922)
Supplement: Supplementary file 2 [file CAM4-8-190-s002.docx]

**Table A. 1** Hodgkin lymphoma patient characteristics

| **HL characteristic** | **GI-HL**  ***n*** (***N*=104)*** | **Esophagus *n* (*N*=30)** | **Gastric  *n* (*N*=34)** | **CRC *n* (*N*=38)** |
| --- | --- | --- | --- | --- |
| **Age of HL diagnosis** |  |  |  |  |
| *Median (interquartile range)* | 30 (22-41) | 31 (25-39) | 26 (21-36) | 30 (22-45) |
| **Treatment period**  1966-1979  1980-1989  1990-2000 | 44 33  27 | 11  12  7 | 11  11  12 | 22  9  7 |
| **HL radiotherapy (RT, primary + recurrence)** | |  |  |  |
| No RT | 6 | 3 | 1 | 2 |
| Mantle field only | 31 | 14 | 6 | 10 |
| Mantle field + infradiaphragmatic RT | 51 | 12 | 24 | 15 |
| Infradiaphragmatic RT only | 11 | 0 | 1 | 9 |
| Yes, field unknown | 5 | 1 | 2 | 2 |
| **HL chemotherapy (CT, primary + recurrence)** | |  |  |  |
| No CT | 22 | 7 | 8 | 7 |
| CT, no procarbazine | 12 | 5 | 1 | 6 |
| Procarbazine-containing CT | 57 | 16 | 20 | 19 |
| *dose <4,2 g/m^2^* | *16* | *5* | *5* | *5* |
| *dose 4,2-8,4 g/m^2^* | *27* | *7* | *13* | *6* |
| *dose >8,4 g/m^2^* | *14* | *4* | *2* | *8* |
| Dacarbazine-containing CT | 7 | 4 | 1 | 2 |
| Yes, type of CT unknown | 13 | 2 | 5 | 6 |
| **HL chemotherapy primary regimens** |  |  |  |  |
| MOPP | 25 | 8 | 6 | 10 |
| MOPP/ABV(D) | 16 | 4 | 7 | 4 |
| ABVD | 3 | 1 | 1 | 1 |
| other/unknown | 17 | 4 | 3 | 10 |
| **HL recurrence** | 43 | 13 | 16 | 14 |
| **HL recurrence chemotherapy** |  |  |  |  |
| MOPP | 12 | 4 | 3 | 6 |
| MOPP/ABV(D) | 10 | 3 | 4 | 2 |
| ABVD | 2 | 2 | 0 | 0 |
| other | 5 | 2 | 1 | 2 |
| **Second non-gastrointestinal cancer diagnoses in HL survivors** † |  |  |  |  |
| Oropharynx | 3 | 2 | 0 | 1 |
| Lung | 1 | 1 | 0 | 0 |
| Breast | 4 | 1 | 2 | 1 |
| Lymphoma (NOS) | 2 | 1 | 1 | 0 |
| Cervix | 1 | 0 | 0 | 1 |
| Connective/soft tissue (NOS) | 1 | 0 | 0 | 1 |

*Abbreviations*: HL, Hodgkin lymphoma; RT, radiotherapy; CT, chemotherapy; NOS, not otherwise specified.

* including two HL patients with a small intestinal cancer.
† GI-HL is the third cancer, excluded from extensive survival analyses.

**Table A. 2** Impact of patient and tumor characteristics on overall survival difference of gastrointestinal cancer in Hodgkin lymphoma survivors (GI-HL) and first primary gastrointestinal cancer patients (GI-1)

| ***Overall survival*** | **Gastrointestinal cancer**  **HR for GI-HL vs. GI-1 (ref)**  **HR 95% CI  *P* value** | | | **Esophageal cancer**  **HR for GI-HL vs. GI-1 (ref)   HR 95% CI  *P* value** | | | **Gastric cancer**  **HR for GI-HL vs. GI-1 (ref)   HR 95% CI  *P* value** | | | **Colorectal cancer**  **HR for GI-HL vs. GI-1 (ref)   HR 95% CI  *P* value** | | |
| --- | --- | --- | --- | --- | --- | --- | --- | --- | --- | --- | --- | --- |
| **Univariable** | 1.30 | 1.03-1.65 | 0.03 | 1.20 | 0.79-1.85 | 0.41 | 1.33 | 0.91-1.96 | 0.15 | 1.36 | 0.90-2.06 | 0.15 |
| **Added characteristic** |  |  |  |  |  |  |  |  |  |  |  |  |
| **Year of incidence** | 1.30 | 1.03-1.64 | 0.03 | 1.20 | 0.78-1.85 | 0.41 | 1.35 | 0.92-1.98 | 0.13 | 1.34 | 0.88-2.03 | 0.17 |
| **Age at diagnosis** | 1.30 | 1.03-1.64 | 0.03 | 1.18 | 0.77-1.83 | 0.45 | 1.37 | 0.93-2.01 | 0.12 | 1.35 | 0.89-2.04 | 0.16 |
| **Gender** male vs. female | 1.30 | 1.03-1.64 | 0.03 | 1.21 | 0.78-1.86 | 0.40 | 1.34 | 0.91-1.97 | 0.14 | 1.36 | 0.90-2.06 | 0.15 |
| **Stage**  I/II vs. III/IV | 1.38 | 1.09-1.74 | 0.008 | 1.14 | 0.74-1.75 | 0.57 | **1.54** | **1.05-2.28** | **0.03** | 1.44 | 0.95-2.18 | 0.09 |
| **Grade of differentiation*** | 1.32 | 1.04-1.67 | 0.02 | 1.20 | 0.78-1.85 | 0.42 | 1.34 | 0.91-1.96 | 0.14 | 1.47 | 0.97-2.24 | 0.07 |
| **Surgery** no vs. yes | **1.51** | **1.19-1.91** | **0.001** | **1.43** | **0.93-2.21** | **0.11** | **1.86** | **1.26-2.75** | **0.002** | 1.35 | 0.89-2.05 | 0.16 |
| **RT** no vs. yes | 1.24 | 0.98-1.58 | 0.07 | 1.14 | 0.74-1.77 | 0.55 | 1.34 | 0.91-1.97 | 0.14 | 1.23 | 0.81-1.88 | 0.33 |
| **CT**  no vs. yes | 1.32 | 1.04-1.67 | 0.02 | 1.08 | 0.70-1.69 | 0.72 | 1.40 | 0.95-2.07 | 0.09 | 1.38 | 0.91-2.09 | 0.13 |
| **Tumor (sub)site**† | 1.32 | 1.04-1.67 | 0.02 | 1.15 | 0.74-1.79 | 0.54 | **1.71** | **1.14-2.55** | **0.009** | 1.29 | 0.85-1.96 | 0.24 |
| **Morphology** AC vs. SCC |  |  |  | 1.11 | 0.72-1.73 | 0.63 |  |  |  |  |  |  |
| ***Disease-specific survival*** |  |  |  |  |  |  |  |  |  |  |  |  |
| **Univariable** | 1.29 | 1.00-1.67 | 0.049 | 1.17 | 0.75-1.84 | 0.49 | 1.43 | 0.95-2.13 | 0.08 | 1.27 | 0.77-2.10 | 0.35 |
| **Added characteristic** |  |  |  |  |  |  |  |  |  |  |  |  |
| **Year of incidence** | 1.29 | 1.00-1.67 | 0.048 | 1.17 | 0.75-1.84 | 0.49 | 1.44 | 0.97-2.16 | 0.07 | 1.26 | 0.76-2.09 | 0.36 |
| **Age at diagnosis** | 1.29 | 1.00-1.67 | 0.049 | 1.16 | 0.74-1.82 | 0.53 | 1.46 | 0.97-2.18 | 0.07 | 1.27 | 0.77-2.09 | 0.36 |
| **Gender** male vs. female | 1.29 | 1.00-1.66 | 0.053 | 1.17 | 0.75-1.85 | 0.49 | 1.43 | 0.96-2.14 | 0.08 | 1.27 | 0.77-2.10 | 0.36 |
| **Stage**  I/II vs. III/IV | 1.37 | 1.06-1.77 | 0.02 | 1.10 | 0.70-1.74 | 0.67 | **1.66** | **1.11-2.49** | **0.01** | 1.38 | 0.83-2.28 | 0.21 |
| **Grade of differentiation*** | 1.31 | 1.01-1.69 | 0.04 | 1.17 | 0.75-1.84 | 0.49 | 1.43 | 0.96-2.14 | 0.08 | 1.36 | 0.82-2.25 | 0.24 |
| **Surgery** no vs. yes | **1.52** | **1.17-1.96** | **0.001** | **1.41** | **0.90-2.22** | **0.14** | **2.00** | **1.33-3.01** | **0.001** | 1.24 | 0.75-2.06 | 0.39 |
| **RT** no vs. yes | 1.24 | 0.96-1.60 | 0.11 | 1.13 | 0.72-1.79 | 0.60 | 1.43 | 0.95-2.13 | 0.09 | **1.13** | **0.68-1.87** | **0.64** |
| **CT**  no vs. yes | 1.32 | 1.02-1.71 | 0.03 | 1.06 | 0.67-1.69 | 0.80 | 1.52 | 1.01-2.28 | 0.045 | 1.31 | 0.79-2.16 | 0.30 |
| **Tumor (sub)site**† | 1.30 | 1.01-1.68 | 0.04 | 1.11 | 0.70-1.76 | 0.67 | **1.80** | **1.19-2.74** | **0.006** | **1.11** | **0.66-1.86** | **0.70** |

Impact of other factors on HR for GI-HL vs. GI-1 (ref), indicated in bold in case of a >10% change.

*Abbreviations*: GI-HL, gastrointestinal cancer in Hodgkin lymphoma survivors; GI-1, first primary gastrointestinal cancer patients; HR, hazard ratio; 95% CI, 95% confidence interval; AC, adenocarcinoma; SCC, squamous cell carcinoma.

* Well-moderate vs. poor-undifferentiated.

† Gastrointestinal cancer: esophagus/gastric vs. small bowel/colorectum, esophageal cancer: upper vs. other, gastric: antrum/pylorus vs. other, colorectal cancer: colon vs. rectum.

**Supplementary figure legend**

**Figure A. 1** CONSORT diagram of gastrointestinal cancer in Hodgkin lymphoma survivors (GI-HL) and first primary gastrointestinal cancer patients (GI-1)

* The Netherlands Cancer Registry (NCR) attempted to register previous cancers for all patients with incident cancer since 1989; the history of previous cancers is known to be incomplete.
